# Influence of the Ag Content on the Natural and Thermal Induced Oxidation of Cu Thin Films

**DOI:** 10.3390/ma17235974

**Published:** 2024-12-06

**Authors:** Maria C. Carrupt, Ana P. Serro, Ana P. Piedade

**Affiliations:** 1 University of Coimbra, CEMMPRE, Department of Mechanical Engineering, 3030-788 Coimbra, Portugal; m.cristina_borges@hotmail.com; 2 University of Lisbon, Instituto Superior Técnico, CQE, Department of Chemical Engineering, 1349-017 Lisbon, Portugal; anapaula.serro@tecnico.ulisboa.pt

**Keywords:** Cu/Ag thin films, preferential crystallographic orientation, natural and thermal-induced oxidation, energy saving systems

## Abstract

In this paper, we studied the deposition and characterization of monolithic and silver-doped copper coatings using RF magnetron sputtering. The main objective was to examine the impact of different Ag contents on natural and thermally induced aging when compared with monolithic copper coatings. For this purpose, the as-deposited surfaces were left exposed to normal temperature and humidity conditions during one year (natural) and were annealed at 200 °C in a non-controlled atmosphere. To evaluate the results of these treatments, the films were characterized in terms of surface and cross-section morphology, structure, chemical composition, wettability, and surface energy. The as-deposited monolithic copper films exhibit a clear face-centered cubic structure with a very strong preferential crystallographic orientation according to the (111) diffraction plane. The presence of Ag in the as-deposited coatings decreased the ability of the films to be wetted, increasing their hydrophobicity and jeopardizing crystallographic orientation development according to the (111)-Cu diffraction plane, particularly after annealing, when compared to Cu films. Through annealing, Cu_2_O and Ag_2_O were formed, leading to a significant decrease in surface energy and reduced wettability. These results can help elucidate and estimate the life span of smart windows, batteries, and solar panels, which are some of the many applications for these coatings.

## 1. Introduction

In recent years, copper and silver coatings have been attracting more and more attention from researchers because of their known thermal, electrical, optical, and chemical properties. Their set of properties allows their application in different areas but, more recently, the focus has been on increasing performance in battery technologies [1,2].

Ag-based alloys have been tested as catalysts to substitute costly Pt-based electrocatalysts for the oxygen reduction reaction (ORR) in alkaline media. Binary thin films, such as CuAg, deposited by PVD with well-controlled compositions, were developed to evaluate the oxygen reduction reaction mechanism on Ag surfaces. CuAg has an overall superior ORR activity than pure Ag, in addition to faster ORR kinetics at low overpotentials, surpassing pure Ag at higher overpotentials. An increase in the activity of Ag-based catalysts for the ORR is important for improving the performance and economic outlook of alkaline-based fuel cell and metal−air battery technologies [1,2]. Ag-based electrocatalysts are also considered to be the most promising catalysts for CO production, via CO_2_ electrocatalysis reduction (CO_2_RR) on a large scale, due to their relatively higher catalytic performance [3,4,5]. Results of the application of bimetallic catalysts, with a different Cu/Ag ratios and doped with CeO_2_ NRs (CuAg/CeO_2−x_), indicate an electron delocalization effect, resulting in an electronic structure change and the formation of the highest concentration of oxygen vacancies, promoting CO_2_ adsorption and activation as well as facilitating the formation of COOH [4].

Solid-state batteries (SSBs) for electrical vehicles are a great promise. However, the assembly of pure Li metal with solid electrolytes (SEs), with low SE–Li interfacial resistance, has been a challenge due to high Li reactivity. To overcome this problem, the “Li-free” cell concept, in which the Li metal anode is electroplated in situ on the current collector (CC), represents an opportunity. In this context, LIZO (Li_7_La_3_Zr_2_O_12_) is an appealing electrolyte to develop for this battery architecture, due to its high ionic conductivity and excellent stability against Li metal. To avoid the growth of lithium as a film-like anode, which leads to premature short circuits, Fallarino et al. [6] developed an artificial interface of a nanometer-thick Ag–Cu bilayer deposited directly onto LIZO by sputtering, forming a Li/LLZO/Ag cell (100 nm)/Cu (600–900 nm), to minimize the interfacial resistance. The formed alloy of Ag–Li promoted the more homogeneous deposition of Li onto Cu because the Ag–Li layer acts as a seed layer for Li nucleation, allowing for the control of Li deposition.

Considering a completely different application, it is known that in recent decades, researchers have been looking for the best way to reduce energy consumption. The development of smart windows promises that a reduction in heat transfer between indoor and outside environments will bring indoor comfort. In this area, metallic materials such as Ag, Au, Cu, and Al have been used as metallic layers in the dielectric–metal–dielectric (DMD) structure, because of their low emission [7,8]. To assemble the DMD, thin films for transparent heat regulation (THR) are deposited by sputtering. The thickness and the crystalline structures are very important for efficiency [7,8,9]. The DMD composed of TiO_2_/Cu/TiO_2_ has been widely tested, and heat reflection with a peak visible transmittance of around 80% and IR reflectance of 60% was achieved [8]. Different research groups have also investigated DMDs composed of TiO_2_/Ag/TiO_2_. Silver possesses the lowest optical losses across a very broad spectral range, spanning from the blue to the near-IR wavelengths. Consequently, Ag is the metal of choice in modern Low-E (low-emission) coatings [9,10,11,12,13,14].

In another field, some authors have also considered using metallic thin films as coatings in the inner cavity of molds for the injection of plastics to increase the cooling rate and ensure the amorphous structure of the polymers. Metallic thin films are also applied onto the cavity of molds as an interlayer for ceramics coating [15].

Regardless of the use, these metallic coatings are subjected to environmental conditions, a natural aging process, or high temperatures, which lead to oxide formation, which impacts the thermal, electrical, and optical properties and also influence surface characteristics such as wettability and the surface energy of metallic thin films. Additionally, temperature exposure can promote changes in microstructure, including crystallographic orientation and grain size [8,16,17,18,19,20,21]. Consequently, it is significant to recognize the influence of natural and thermally induced aging on these coatings.

In this work, thin films of copper and silver-doped copper were developed, aged (both naturally and by thermal treatment), and characterized in terms of microstructure, morphology, chemical composition, wettability, and surface energy. These films were deposited by magnetron sputtering, thermally treated by annealing at 200 °C for 30 min to mimic the temperature of injection molding of amorphous polymers, and also naturally aged by exposure to air at room temperature for 1 year. Some of the films were also thermally annealed and then left at room conditions for 1 year. The published research on thin film aging usually focuses on annealing at different times and temperatures or observing changes over days or months of exposure to air at room temperature. To the best of the authors’ knowledge, no work has been reported with the experimental approach reported here.

## 2. Materials and Methods

### 2.1. Depositions and Annealing of Thin Films

Thin films of copper and silver-doped copper were deposited on glass, silicon, and steel AISI P20. Coated glass and silicon were used for the characterization of microstructure by X-ray diffraction (XRD), surface energy through contact angle measurements, and morphology by scanning electron microscopy (SEM). Micro-Raman analysis was performed in coated steel. Before deposition, all substrates were ultrasonically cleaned in acetone and alcohol for 10 min in each liquid. The reason for using very different materials as substrates is related to the type of characterizations that need to be performed: contact angles require a surface with constant and very low roughness, such as glass; DRX needs monocrystalline materials, such as Si, so the substrate diffraction peaks do not interfere with the evaluation of the diffractograms; steel was used as the substrate for the micro-Raman analysis because metals do not have any peaks in Raman spectroscopy and, consequently, do not interfere in the interpretation of the obtained spectra.

**Figure 1:**
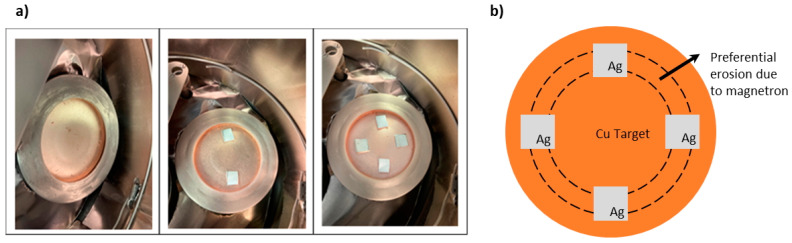
(**a**) Real target configuration used for deposition. From left to right: copper target, copper target with two pieces of silver, and copper target with four pieces of silver. (**b**) Schematic representation of the “cardinal” positions of the Ag pieces over the preferential eroded area.

The films were deposited by RF magnetron sputtering (13.56 MHz), with water cooling of the substrate holder. Due to this very particular characteristic of the sputtering equipment, the differences induced in the coating properties due to different materials being used as substrates is completely attenuated. The equipment we used was the Edwards Coating System E306A (Edwards, Lisbon, Portugal), with power sources of 1000 W and 500 W branched to the target and substrate, respectively. A target of 99.99% pure copper, with 100 mm of diameter and 3 mm of thickness, was used. For the deposition of copper films with silver, silver pieces (10 × 10 mm^2^) with 99.99% purity were placed onto the copper target over the area with higher erosion, as shown in Figure 1. The silver pieces were placed over the preferential site of erosion of the target due to the presence of the round magnetron beneath it. This approach allows to vary the chemical composition of the coatings without the need to buy separated specific alloyed targets, therefore making it a much more viable approach economically.

Deposition was carried out in a non-reactive atmosphere using argon with 99.9999% purity. After placing the substrate holder into the chamber, it was evacuated until an ultimate pressure of 1 × 10^−4^ Pa. Before deposition, both the target and substrates were plasma-cleaned for 5 min at 150 W and 0.7 Pa. A shutter was placed between the target and substrates to avoid cross-contamination. Three types of thin films were deposited: (i) pure copper (Cu); (ii) Ag-doped Cu, co-deposited with two pieces of silver (Cu_2Ag); and (iii) Ag-doped Cu, co-deposited with four pieces of silver (Cu_4Ag). All deposition was carried out under the same parameters: a deposition pressure of 0.7 Pa and a deposition power of 400 W, for 10 min.

**Figure 2:**
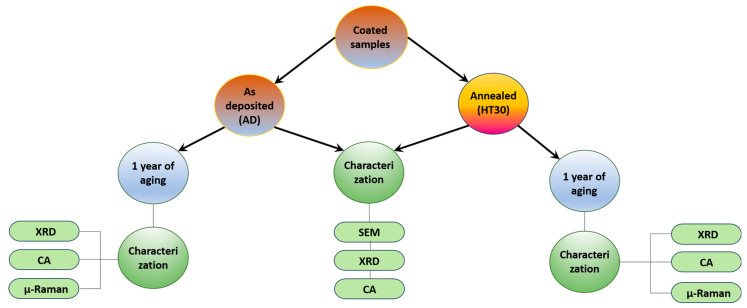
Schematic representation of steps for preparation and characterization of samples.

The coated substrates were divided into two main groups: in the first one, they were kept as-deposited (AD), and those of the second group were annealed for 30 min at 200 °C (HT30). These two groups were subdivided as follows (Figure 2): half of the samples were immediately characterized by SEM, XRD, and contact angles (CA), and the other half was kept exposed to air for 1 year. After 1 year, the surfaces were characterized by XRD, CA, and micro-Raman.

### 2.2. Characterization Techniques

The surface and cross-section morphologies, as well as the qualitative and semi-quantitative chemical composition, were evaluated by SEM in secondary electron (SE) mode, using FEI QUANTA 400 FEG ESEM equipment (FEI, Munich, Germany). The thickness of each coating was determined using the software Image-J 1.52a from the obtained micrographs. Energy-dispersive spectroscopy (EDS) was performed in an Edax Genesis X4M coupled to the SEM equipment (EDAX, LLC, Pleasanton, CA, USA).

The structure was characterized by XRD, using an X’Pert PRO (PANalytical, Amsterdam, The Netherlands) apparatus with a cathode of Cu (λKα1 = 0.154060 nm), a current of 40 mA, and a voltage of 45 kV. The analyses were carried out at room temperature, in conventional mode, between 25° and 95°, with a step of 0.02°, and an incident slit maximum of 1° to improve the analysis area. The peaks in the XDR diffractogram were identified according to reference patterns from the International Centre for Diffraction Data (ICDD): 00-004-0836 (Cu), 00-001-1164 (Ag), 00-001-1142 (Cu_2_O), and 01-072-2108 (Ag_2_O).

The chemical composition was also evaluated by micro-Raman spectroscopy to confirm oxide identification. The spectra were collected in a Renishaw in Via Reflex Microscope coupled to a Leica microscope (DM2700 M) (Leica, Famalicão, Portugal, at room temperature, in a 100–1000 cm^−1^ spectral range, with a 532 nm laser line, and the spectral resolution was 2 cm^−1^ using an objective lens with ×50 of magnification. The laser intensity was kept at 10% of the maximum power (50 mW) during 10 s of exposure. The software from Renishaw’s (Wotton-under-Edge, UK) WiRETM 1.0 was used to acquire the spectra and analyze all samples.

The wettability characteristics of the surfaces were assessed by measuring the static contact angle of five drops of Milli Q water, each with 5 µL volume, in Biolin Scientific Attension Theta Flex equipment (Paralab, Porto, Portugal) using the sessile drop method. The surface energy (ϒs) of the samples was calculated using the polar (ϒ_s_^p^) and dispersive (ϒ_s_^d^) components of Milli Q water and formamide according to the procedure previously described in the literature [22]. For this calculation, in each sample, the contact angles of five drops (5 µL each) of water and five drops of formamide were measured.

## 3. Results and Discussion

### 3.1. SEM and EDS Characterization

**Figure 3:**
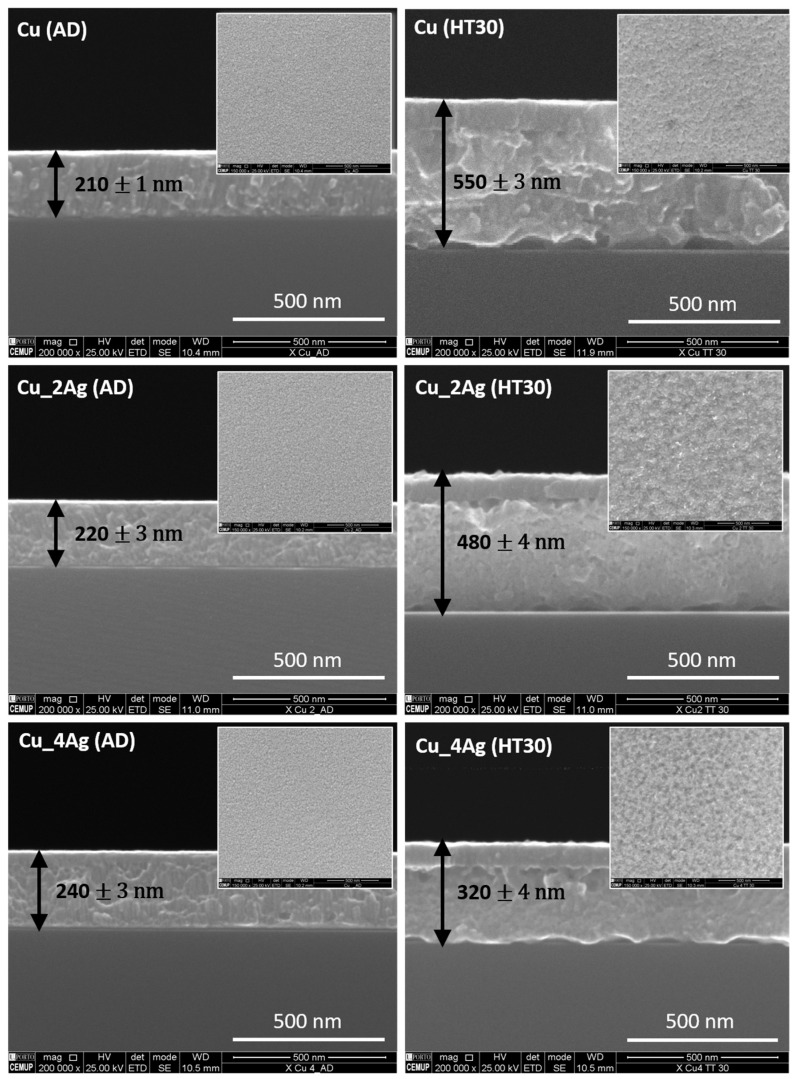
SEM micrographs of the cross-section of the as-deposited (AD) films and films annealed at 200 °C (HT30) on the high side, with the surface micrographs in detail.

The SEM micrographs of the surfaces and cross-sections are shown in Figure 3. The EDS semi-quantitative analysis of the AD Ag-doped Cu thin film (Cu_2Ag and Cu_4Ag) is exhibited in Table 1.

The surfaces in Figure 3 show homogeneous coatings with nanometric grain size, where precipitates are not observed. According to the equilibrium diagram of the Cu–Ag system [23], the atomic percentages of Cu (Table 1), 82.5% and 61.2%, indicate the formation of a solid solution within the domain (α + β). Considering the cooling rate in thermodynamic equilibrium, there is significantly reduced solubility of Ag in Cu (less than 1 at.% at room temperature) [24]. However, these thin films were deposited by sputtering, which allows obtaining metastable structures not predicted by thermodynamics. Additionally, in the particular case of the used equipment, the cooling rate is even higher due to the substrate water cooling system.

Upon comparing the micrographs of the as-deposited (AD) and annealed surfaces, grain size changes are perceptible due to the annealing treatment.

The characterization of the cross-sections shows that the AD films are compact, and their morphology is similar to those represented in Zone T of the Thornton structure zone model [25]. This zone, between Zone 1 (porous structure) and Zone 2 (columnar grains), is a transient zone characterized by a group of poorly defined fibrous grains, which are densely distributed. The thermal treatment produced, in every coating, an even more compact zone on top of the film, which may correspond to the oxidation of the metallic elements. Further characterizations will confirm this possibility, as demonstrated in the subsequent subsections.

The introduction of silver promoted an increase in the thickness of the AD films for the same deposition parameters, ranging from 210 ± 1 nm (Cu) to 220 ± 3 nm (Cu_2Ag) and 240 ± 3 nm (Cu_4Ag).

**Figure 4:**
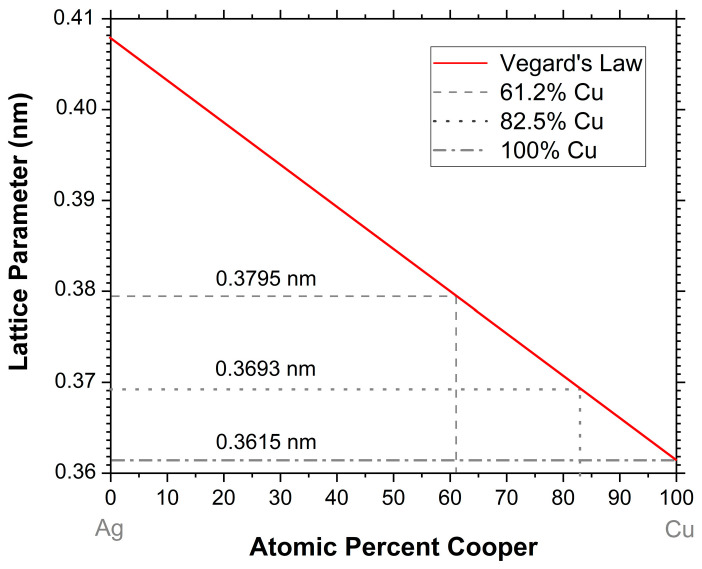
Variation of the lattice parameter of the Cu–Ag system, with the Ag content, in the as-deposited coatings.

These differences can be due to the increase in the copper lattice parameters that occur with the formation of Cu–Ag solid solution [17]. Ag has a higher lattice parameter (0.4179 nm) than Cu (0.36601 nm) and, according to Vergard’s Law, an increase in Ag content increases the lattice parameter of Cu–Ag coatings [23]. The graphic in Figure 4 shows the approximate estimation of the lattice parameter in the Cu–Ag system, according to Vergard’s Law. Plotting the values of the atomic percentage of Cu, determined by EDS (Table 1), the lattice parameter increases as the content of Ag increases. The approximated values of lattice parameters for the thin films, according to Figure 4, are 0.3615 nm (100% Cu), 0.3693 nm (82.5% Cu), and 0.3795 nm (61.2% Cu).

After annealing (HT30), the films exhibit a considerable increase in layer thickness (Figure 3). The monolithic Cu coating experienced the greatest increase (210 ± 1 nm to 550 ± 3 nm), followed by Cu_2Ag (220 ± 3 nm to 480 ± 4 nm), and the film with the highest percentage of Ag (Cu_4Ag) had the smallest increase (240 ± 3 nm to 320 ± 4 nm). These results can originate from two distinct sources.

Firstly, it can be considered that annealing promoted grain growth. Grain growth occurs for Cu–Ag films from a temperature of 180 °C [17], and the growth is controlled by the diffusion of silver atoms to the grain boundary, pinning it and hindering grain growth during heating [8,9]. Since the pin of the grain boundary is associated with the percentage of diffused solute, it is clear why the film of pure Cu exhibited the greatest increase in layer thickness. It has no solute to diffuse and pin the grain boundary during the heat treatment. As the Ag content increases, the amount of diffused solute also increases.

Secondly, the appearance of a dense layer on top of the annealed films can be related to the oxidation of the metals. Considering that Ag has a protective effect because it resists oxidation more than Cu [26], the higher the Ag content, the thinner the oxidation layer will be and, consequently, the smaller the increase in thickness due to the thermal treatment will be.

### 3.2. XRD and µ-Raman Characterization

**Figure 5:**
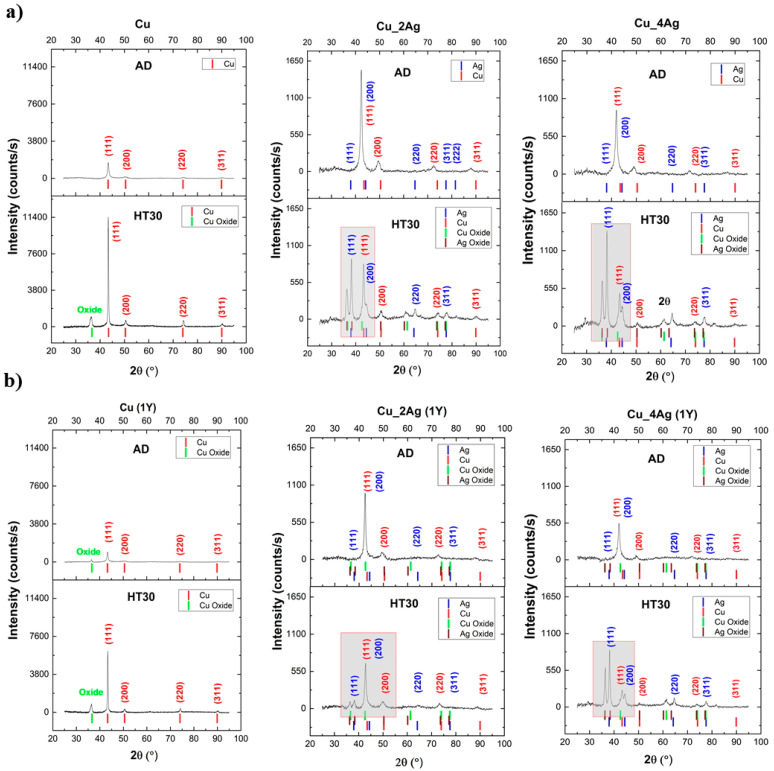
XRD diffractograms of the thin films. (**a**): as-deposited (AD) and after annealing (HT30) coatings; (**b**): films after 1 year of aging (1Y), as-deposited (AD), and with annealing (HT30). The position of the diffraction peaks according to the reference patterns are identified below the curves. The gray zones are zoomed in and presented in Figure 6.

**Figure 6:**
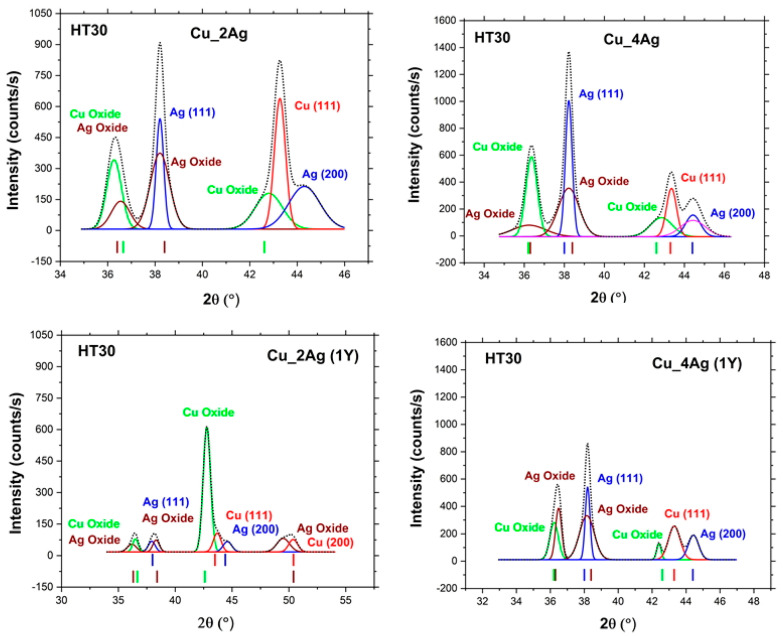
Deconvolution of peaks (111)-Cu, (111)-Ag, and (200)-Ag on Cu_2Ag and Cu_4Ag films subjected to heat treatment at 200 °C for 30 min and aging for 1 year in air.

The coatings—as-deposited, after annealing (HT30), as-deposited + one year of natural aging, and after annealing (HT30) + one year of aging—were analyzed by XRD (Figure 5), and the original positions of the diffraction peaks, according to the reference patterns from ICDD, are identified. The deconvolution of some of the diffraction peaks is presented in Figure 6.

The films have a face-centered cubic (fcc) crystalline structure. The as-deposited (AD) films exhibit preferential crystallographic orientation according to the (111) diffraction plane of copper. The specific sputtering equipment used in the present work favors the growth of coatings according to the lower surface energy plane and higher atomic density, which, in fcc structures, correspond to the (111) diffraction plane [27]. On the films of CuAg, due to the presence of Ag in the lattice parameter, promoting its distortion, the diffraction peaks were shifted to lower angles (2θ) [28], compared with their original position in the reference patterns.

The fact that the nucleation and growth of the films are not according to the thermodynamic equilibrium can promote additional stresses and high dislocation and defect densities [17]. Moreover, microstrains originating from defects can affect the intensity, position, and width of XRD peaks [28].

In the AD coatings, before and after one year of natural aging, the Ag diffraction peaks are not visible, indicating that the silver is either with an amorphous structure [21,29] or coherent with the Cu matrix and cannot be detected using this XRD technique [30]. Annealing promoted a significant increase in the intensity of (111)-Ag for both Cu_2Ag and Cu_4Ag. The increase in the intensity of (111)-Ag is more pronounced on Cu_4Ag, with a higher content of this metallic element, where an increase in the intensity of all silver peaks can be observed. The same results were observed in the literature [29,31], where an increase in temperature during the annealing of thin films led to an increase in the crystallinity of silver, with the corresponding diffraction peaks becoming more intense. Other authors have demonstrated that on films deposited at room temperature, Ag is amorphous [19,21]. Nevertheless, with an increase in the substrate temperature to 300 °C, crystalline Ag is identified in the XRD diffractogram [19,21]. Based on the results in the literature, it can be inferred that silver in AD films is amorphous and remains amorphous even after one year of aging.

Annealing also promoted the oxidation of the films. The oxides were very well-identified by the pattern code (00-001-1142) for copper oxide and the pattern code (01-072-2108) for silver oxide, both with more than three identifiable peaks. The oxides that formed are Cu_2_O and Ag_2_O. These results confirm the observation made in the SEM cross-sections micrographs. Figure 6 shows the peaks of Cu_2_O and Ag_2_O before deconvolution.

**Figure 7:**
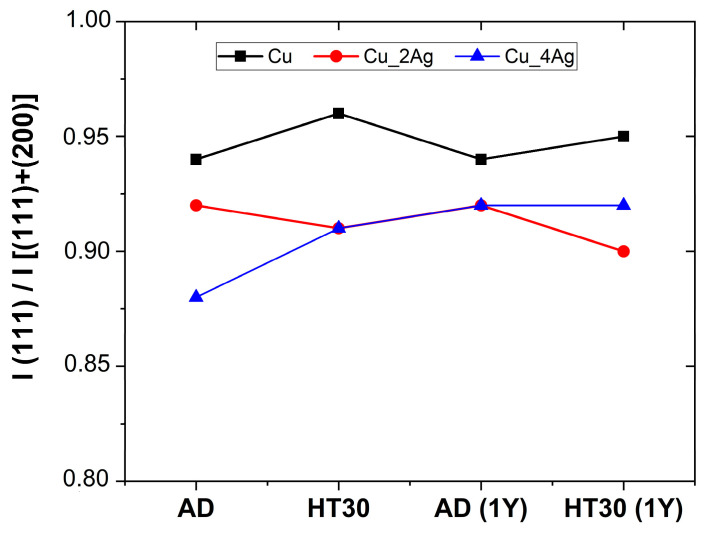
Influence of heat treatment and natural aging on intensity ratio I(111)/[I(111)+I(200)], considering only crystallographic planes of Cu.

The original positions of the Cu_2_O peaks, according to the pattern code, are 2θ = 36.7°, 2θ = 42.6°, 2θ = 61.4°, and 2θ = 74.0°. Due to the effects of heat treatment and aging, these peaks in Cu_2Ag and Cu_4Ag diffractograms are shifted around these positions. Similar positions of 2θ = 36.7° and 42.6° were observed by Messaoudi et al. [32]. They evaluated the influence of the annealing temperature on the decomposition of Cu_2_O into CuO, and for temperatures lower than 300 °C, the presence of CuO was not detected. For temperatures higher than 350 °C, diffraction peaks corresponding to CuO appeared. Besides the coincidence of the 2θ position in Figure 7, their results reinforce that in this work, since the heat treatment has been carried out at 200 °C, the peaks identified are Cu_2_O and cannot be CuO.

According to the pattern code (01-072-2108), the peaks of Ag_2_O appear at the positions 2θ = 36.8°, 2θ = 38.4°, 2θ = 50.4°, 2θ = 60.2°, and 2θ = 73.7°. The peak 2θ = 38.4° was also identified by Wterahouse [33] as Ag_2_O, after annealing at 200 °C. After evaluating the decomposition of silver oxides during heat treatment at temperatures between 100 °C and 400 °C, their results show that for temperatures above 200 °C, this peak (2θ = 38.4°) corresponds to Ag_2_O. Below 200 °C, this peak corresponds to AgO, indicating the complete thermal reduction of AgO to Ag_2_O.

With heat treatment (HT30), in the pure Cu coating, the intensity of the (111) diffraction peak increases significantly, while in Cu_2Ag and Cu_4Ag, the intensity of the same Cu diffraction peak decreases.

The degree of preferential orientation can be evaluated by the relative ratio of the (111) diffraction peak intensity in relation to the sum of the most intense diffraction peaks according to the reference patterns, i.e., (111) and (200) [I (111)/I (111) + I (200)] [17,27]. The results of this ratio were calculated for the diffraction peaks of Cu and the result is shown in Figure 7. The increase in the crystallinity of silver promoted reorganization in the crystalline structure of the films, which is reflected in the intensity of the peak (111)-Cu. The presence of Ag hindered the development of preferential crystallographic orientation in the silver-doped copper films. The same result was obtained by researchers for a Cu–Ag alloy film [34].

**Figure 8:**
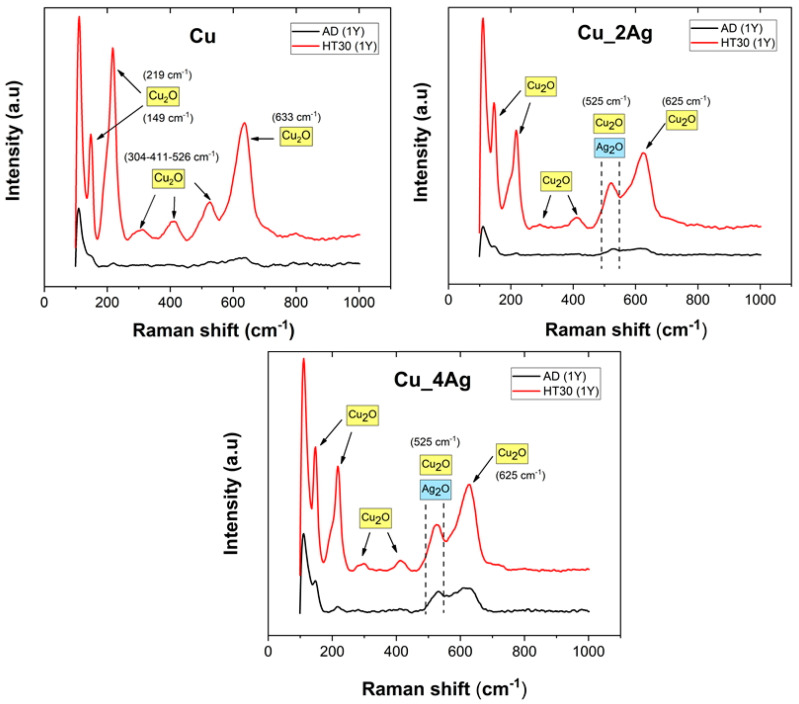
Raman spectra of as-deposited and annealed films after 1 year (1Y) of aging.

Micro-Raman analysis was performed on the natural and thermal-treated aged thin films (Figure 8) to confirm the presence of Cu_2_O and Ag_2_O. The graphics evidenced that the oxides that began forming during heat treatment evolved strongly during the following natural aging period, resulting in well-defined peaks, with high intensity, compared with the as-deposited films after natural aging (AD(1Y)). Copper oxides are easily detected by Raman. The Cu film HT30 (1Y) presents peaks at 149, 219, 304, 411, 526, and 633 cm^−1^. According to the literature, Cu_2_O exhibits peaks at 145, 220, 297, 411, 492, 633, and 786 cm^−1^ [35], as well as 525 cm^−1^ [36]. The coatings of silver-doped copper HT30 (1Y) show peaks at the same Raman shift of the Cu film, and these were assigned to Cu_2_O.

The Raman spectrum of Ag_2_O has a band at 490 cm^−1^ due to symmetry considerations [34,37]. A band at 525 cm^−1^ was assigned to Raman vibration after reducing AgO to Ag_2_O at 200 °C [34], similarly to the band at 530 cm^−1^ [37]. The overlay of several parts of the spectra corresponding to the oxides is expected, as they both share a peculiar cubic cuprite structure, made of two interpenetrating lattices, one fcc of metal atoms (Ag or Cu) and another of body-centered cubic (bcc) oxygen atoms (O) [34,38]. Therefore, we can infer that the band of Ag_2_O overlaps with the band of Cu_2_O between 450 and 550 cm^−1^.

Additionally, although metals do not present any signal in Raman spectra, if oxidized, they can present some peaks. However, those are not present in the present spectra because (i) if those oxides were formed during thermal annealing, their appearance would contribute to the increase in tension at the substrate/coating interface, resulting in the detachment and loss of integrity of the coating, which was not the case; (ii) oxides of low-alloy steel, such as PI20, appear at 670 cm^−1^ in Raman spectra, according to the literature [39,40], which does not correspond to the observed peaks in this work.

The spectra of Cu and silver-doped copper, AD (1Y), are similar, which can be explained by the fact that some authors, as explained before, consider silver to have an amorphous structure. Moreover, from the thermodynamic perspective, the formation enthalpy of Cu_2_O (−168.6 kJ/mol) favors its formation instead of Ag_2_O (−31.0 kJ/mol) [41].

### 3.3. Contact Angles

**Figure 9:**
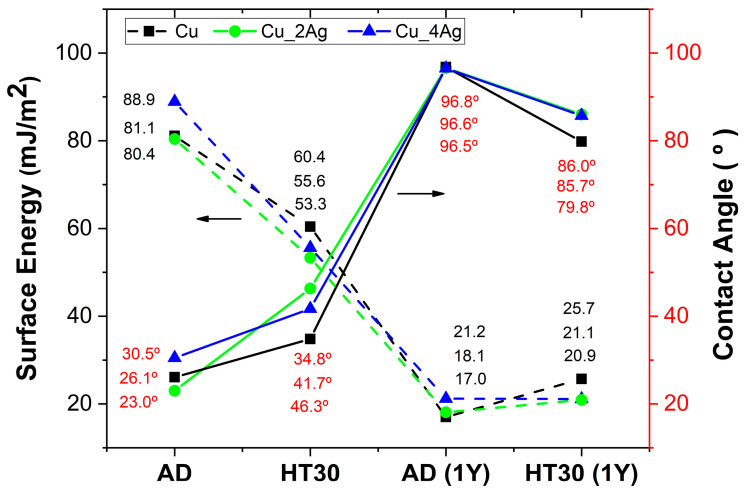
The surface energy (dashed lines) and water contact angles (solid line) of the studied surfaces (the lines serve only as a visual aid).

The contact angles were measured to evaluate the effects of annealing and aging on the wettability and surface energy of the deposited films. The results are plotted in Figure 9, where the values of surface energy (black) and contact angles (red) are reported.

Comparing the surface energy before and after one year of natural aging (1Y), the oxidation process promoted a decrease in surface energy as expected, since the surface energy of ceramic materials (in this case, oxides) is lower than that of metals. Although the surface energy values of the AD coatings are the highest of the studied systems, they are lower than was expected for bulk pure Cu (1810 mJ/m^2^) or pure Ag (1310 mJ/m^2^) [42]. The main reason that can justify this behavior is the fact that it is a coating, not a bulk material, produced by a technique that does not follow thermodynamic laws. Moreover, and as previously referred to, the preferential orientation according to the diffraction plane with lower surface energy contributes to the low values of surface energy when compared with bulk materials.

After annealing (HT30), the contact angles increased, indicating a less reactive surface, and, consequently, an decrease in the surface energy values. A similar result was also observed by other authors [43], who attributed the variation to the increase in grain size due to thermal treatment. The XRD diffractogram (Figure 5) shows a decrease in the intensity of the Cu (111) diffraction peak and an increase in the intensity of the Ag (111) diffraction peak, due to the increased degree of crystallinity of Ag, as explained earlier. Moreover, the formation of a surface oxide layer with less reactive materials, oxides, justifies the observed results.

One year after thermal treatment, the surface energies are very similar to the values of the AD after one year of natural aging. This may indicate that HT30 surfaces, although oxidized, have not attained the most stable chemical configuration, and, therefore, evolve to more stable oxides. This is a natural process, as all systems (including surfaces) tend to diminish the entropy [44].

The surfaces of the coatings thermally treated after deposition (HT30) are the only ones with pronounced hydrophilicity. All the other surfaces present contact angles around 90°, which makes it difficult to classify them as hydrophilic or hydrophobic because the values are in the limit zone.

## 4. Conclusions

Thin films of copper and silver-doped copper were deposited on glass and silicon by PVD (RF magnetron sputtering), annealed at 200 °C, and aged for one year in the air. The effects of Ag content, annealing temperature, and aging were evaluated. The films are crystalline with an fcc structure and a preferential crystallographic orientation according to the (111) diffraction peak. However, XRD analysis indicated that in as-deposited films, it is probable that Ag is in an amorphous state, and only after annealing does it become crystalline.

SEM images show that the presence of Ag promoted an increase in the thickness of the as-deposited thin films. During annealing, Ag restricted the increase in film thickness by restricting grain growth. The addition of Ag also reduced the wettability of the films and hindered the degree of preferential crystallographic development, namely after annealing.

The annealing temperature, besides stimulating the crystallization of Ag, promoted the formation of the oxides Cu_2_O and Ag_2_O. Annealing increased the contact angles of the films with water and consequently decreased the surface energy, which turned the surfaces less chemically reactive. After annealing, and during the subsequent year, the oxidation process continued, resulting in more reduction in the surface energy.

The obtained results indicate that it is necessary to evaluate the variation in the thermal properties (from conduction to insulation) of the studied metallic coatings to evaluate the variation in the performance of these type of coatings when used in energy saving applications, such as the ones mentioned in the introduction.

## Figures and Tables

**Table 1:** Semi-quantitative chemical analysis, as atomic (at.%) and weight (wt.%) percentages, of the Ag-doped Cu coatings, determined by EDS.

Coatings	Chemical Composition			
	(at.%)		(wt.%)	
	Cu	Ag	Cu	Ag
Cu_2Ag	82.5	17.5	67.6	26.8
Cu_4Ag	61.2	38.8	47.4	51.0

## Data Availability

The original contributions presented in this study are included in the article. Further inquiries can be directed to the corresponding author.
